# Influence Mechanism of Ageing Parameters of Cu-Cr-Zr Alloy on Its Structure and Properties

**DOI:** 10.3390/ma15217605

**Published:** 2022-10-29

**Authors:** Yuxia Ma, Huiqin Chen, Hui Li, Shue Dang

**Affiliations:** 1 School of Materials Science and Engineering, Taiyuan University of Science and Technology, Taiyuan 030024, China; 2 Heavy Castings and Forgings Engineering Technology Research Center of Shanxi Province, Taiyuan 030024, China; 3 Taiyuan Jinxi Chunlei Copper Co., Ltd., Taiyuan 030003, China

**Keywords:** Cu-Cr-Zr alloy, precipitated phase, orientation relationship, tensile strength, electrical conductivity

## Abstract

The Cu-1.0% Cr-0.1% Zr alloy in a solid solution state was investigated by ageing treatments at different temperatures and holding times. The structure and performance were characterized and tested by using X-ray diffraction (XRD), a transmission electron microscope (TEM), a universal material testing machine, and an eddy conductivity detector. The influence laws of ageing temperature and the holding time on the structures and properties of the Cu-Cr-Zr alloy were analyzed. Results demonstrated that, with the increase in ageing temperature and holding time, the percentage and size of the Cr precipitated phase increased, and the dislocation density decreased. The tensile strength first increased to the peak value and then decreased. The electrical conductivity increased and the amplitude decreased. The tensile strength of the alloy reached the peak (359 ± 2 MPa) after ageing at 450 °C for 60 min, and the electrical conductivity was 91.9 ± 0.7% IACS. In addition, in the ageing precipitation process, the chromium precipitated phase had face-centered cubic structure (FCC) and body-centered cubic structure (BCC) structures, and the FCC Cr phase can be transformed into a BCC Cr phase. FCC Cr, BCC Cr, and Cu_3_Zr precipitation phases maintained different orientation relationships with the Cu substrate.

## 1. Introduction

Copper (Cu) and copper alloys have been widely used in electrical connectors, resistance welding rods, welding rod continuous casting mold liners, integrated circuit lead frames, and other devices owing to their excellent electrical conductivity, thermal conductivity, and corrosion resistance [1,2,3,4,5]. The rapid development of technical industries, such as the electrical engineering, aerospace, and electronics industries, has led to higher requirements in terms of the performance of Cu alloys, especially in their strength and electrical conductivity. Therefore, elements with extremely low solubility are added to Cu and Cu alloys to improve their strength and conductivity through precipitation strengthening. The Cu-Cr-Zr alloy is a type of high-strength and high-conductivity Cu alloy that has received significant research attention. Adding Cr and Zr simultaneously to Cu alloys can improve heat resistance, abrasive resistance, and strength, as well as increase the anti-softening temperature without a loss of electrical conductivity. Furthermore, Cr and Zr interact well; therefore, refining the Cr precipitated phases can further improve the strength of the alloy [6,7,8,9].

The high strength of the Cu-Cr-Zr alloy can mainly be attributed to precipitation strengthening. High-density Cr nano-precipitated phases are produced in the alloy after ageing treatment, which improves its mechanical properties [8,10,11]. The high electrical conductivity is mainly attributed to the decreased concentration in solute atoms in the solid solution of the Cu substrate during the ageing treatment [12,13,14]. The volume fraction of the precipitated phases determines the electrical conductivity of the alloy [15]. Given the high volume fraction of the precipitated phase, there are fewer solute atoms in the substrate, a lower probability of electron scattering, and a higher electrical conductivity [16]. Therefore, the electrical conductivity and mechanical properties of the Cu-Cr-Zr alloy are determined according to their structures after thermal treatment.

The crystal structures of the precipitated phase of the Cu-Cr-Zr alloy and its orientation relationship with the Cu substrate have been studied since the 1970s. For example, Zeng et al. [17,18] studied and calculated the phase diagrams of the Cu-rich zone of the Cu-Cr-Zr alloy system, and found Cr, Cr_2_Zr, Cu_5_Zr, and Cu_3_Zr phases in the alloy. Additionally, Kuznetsov et al. [19] systematically calculated the vertical section phase diagram of Cu-Cr-Zr alloys with 0.5%, 1.5%, and 5% Cr contents and the results showed that there were Cr, Cu_3_Zr, Cu_51_Zr_14_, Cu_8_Zr_3_, and Cu_10_Zr_7_ phases in the alloys. Cheng and Peng et al. [14,20] determined the precipitation order of Cr phases in the Cu substrate as follows: Supersaturated solid solution; Guinier–Preston (GP) zone; FCC Cr phase ordering; BCC Cr phase. Knight et al. [8] found that some metastable state FCC Cr phases were also precipitated. In terms of the crystal structure of the Cr precipitated phases, some researchers [21] proposed that the precipitated phases were steady-state BCC Cr phases under over-ageing conditions. Fujii et al. [9] found that BCC Cr phases were transformed from FCC Cr phases. Chbihi et al. [22] also believed that there were two structures of Cr phases (BCC and FCC) in the Cu substrate and that BCC Cr phases were precipitated through the nucleation of FCC Cr phases. Peng et al. [23] believed that the precipitation order of Zr phases was as follows: Supersaturated solid solution; Zr atomic cluster; CuCrZr phases; Cu_5_Zr phases. Furthermore, many scholars [1,24,25,26] have shown that Zr atoms formed Cu_5_Zr phases in the Cu substrate during the ageing precipitation of the alloy. Su et al. [27] pointed out that there were transgranular ordered CrCu_2_ (Mg, Zr) (DO3) precipitated phases in the Cu-Cr-Zr-Mg alloy. Liu and Huang et al. [13,28] believed that the existing ordered phase at precipitation included metastable Heusler phases (CrCu_2_Zr phases), which were then decomposed into BCC Cr phases and Cu_4_Zr phases with an orthogonal structure through the follow-up ageing process. Furthermore, some researchers found that the Zr precipitated phase is the Cu_51_Zr_14_ phase [6].

Nevertheless, there still is no consensus regarding the precipitation order of Cr phases during ageing treatments, in addition to which types of Zr phases are obtained. Therefore, it is imperative to further study the crystallographic characteristics and orientation relationship with the substrate during the precipitation of the Cu-Cr-Zr alloy, which can facilitate the improvement of the performance of the alloy. Therefore, the structural evolution of alloys after ageing at different ageing temperatures and holding times was discussed in this study. Furthermore, their mechanical properties and electrical conductivities after ageing were also analyzed.

## 2. Materials and Experimental Methods

Five millimeter thick Cu-1.0% Cr-0.1% Zr (Fe, 0.05% max; Si, 0.1% max; others, 0.2% max; wt%) alloy plates after solid-solution treatment at 950 °C for 2 h were used in this study. Then they were treated by ageing at different temperatures (300 °C, 350 °C, 400 °C, 450 °C, and 500 °C) for different times (15 min, 30 min, 60 min, 90 min, and 120 min).

The mechanical properties of the samples were tested using an AG-X100KN Shimadzu electronic universal material testing machine (Shimadzu, Kyoto, Japan). Dog bone-shaped samples were used in the tensile test, the samples’ total size was 145 × 25 mm, the parallel part was 80 mm in length, and the width was15 mm. Measurements were carried out at the extension rate of 0.02 mm/min under the temperature of 25 ± 2 °C thrice and the average value was collected. The electrical conductivity was tested using the SMP10 eddy electrical conductivity tester (Fischer, Stuttgart, Germany) and the diameter of the probe was 14 mm. Three measurements were performed at different positions of the same sample and the average value was chosen. The lattice strain of the samples was determined by using an X-ray diffraction (XRD, DX2700B, Haoyuan Instrument, Dandong, China) instrument. On this basis, dislocation density was calculated by the following Equation (1) [29,30,31,32]. In XRD, a Cu target (wavelength = 0.15406 nm) was used, and the scanning rate was 2 °/min. The angle range was 20–100°. Structures of the alloy after the ageing treatment were characterized by transmission emission microscope (TEM, JEM F200, JEOL, Tokyo, Japan), including a bright-field image, high-resolution transmission electron microscope (HRTEM), selected area electron diffraction (SAED), and inverse fast Fourier transform (IFFT). Samples for the TEM observation were thinned to less than 30 μm through mechanical grinding and then placed into a nitric acid-methanol solution (CH_3_OH:HNO_3_ = 7:3). Finally, the samples were thinned using an electrolytic double jet thinning instrument (DJ2000, Hisky Technology, Beijing, China) under a temperature of −30 °C and a voltage of 20 V.

(1) $$\rho =16.1\times \frac{\varepsilon^{2}}{b^{2}}$$

where ρ is the dislocation density, *b* is the Burgers vector of dislocation and ε is the microstrain obtained by Hall–Williamson’s method.

## 3. Results

**Figure 1:**
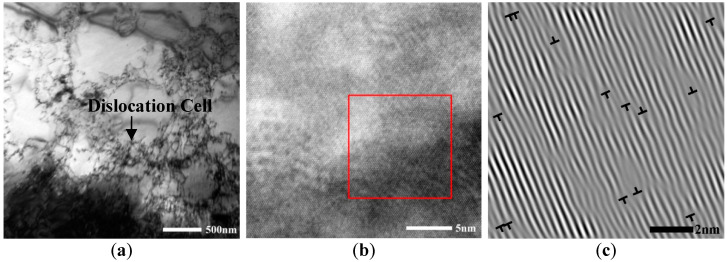
TEM of the alloy after solution treatment: (**a**) Bright-field image; (**b**) HRTEM image; (**c**) IFFT image of the red selected area in (**b**).

The TEM structures of the Cu-1.0% Cr-0.1% Zr alloy after solid-solution treatment are shown in Figure 1. The bright-field images of TEM are shown in Figure 1a and the HRTEM is shown in Figure 1b. Several dislocations were present in the alloy structure, which combined into dislocation cells. According to the IFFT image (Figure 1c), several unevenly distributed misfit dislocations were observed. Additionally, the dislocation density in the alloy was found to be 2.528 × 10^15^ m^−2^ and the lattice constant was 0.45448 nm, which was higher than that of Cu (0.3615 nm). The lattice distortion was severe; under these circumstances, the tensile strength of the alloy was found to be 256 ± 4 MPa and its electrical conductivity was 34.5 ± 0.3% IACS.

### 3.1. Influence Laws of Ageing Temperature on Ageing Structures

**Figure 2:**
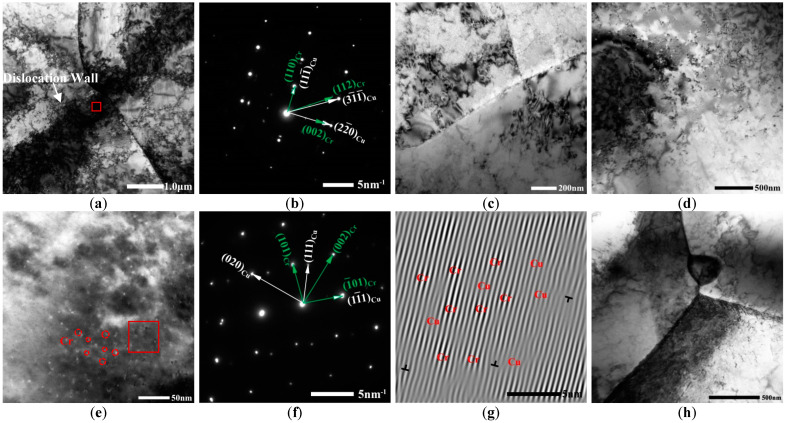
TEM image after 30 min ageing at different temperatures: (**a**) 300 °C; (**b**) SAED of the square area in (**a**); (**c**) 350 °C; (**d**) 400 °C; (**e**) 450 °C; (**f**) SAED of the square area in (**e**); (**g**) IFFT image of the square area in (**e**); (**h**) 500 °C.

The TEM images of the Cu-Cr-Zr alloy after the ageing treatment after 30 min at different temperatures are shown in Figure 2. With increases in the ageing temperature, there were more precipitates in the ageing structure but fewer lattice distortions and a lower dislocation density. The specific lattice distortion and dislocation density are shown in Table 1. According to the SAED using [112]_Cu_ as the zone axis after ageing at 300 °C, other than the reflection of the substrate, there was a precipitation of [$\bar{1}10$]_Cr_ phases coherent with the substrate (Figure 2b) and a dislocation wall formed by the involvement of dislocations in the substrate (Figure 2a). After ageing at 400 °C, the number of precipitates in the substrate increased obviously, most of which were at positions that had a high dislocation density (Figure 2d). HRTEM images after ageing at 450 °C are shown in Figure 2e. Obviously, several fine dispersed secondary phases were precipitated in the substrate. It can be seen from the SAED (Figure 2f) results that the precipitated phase was the Cr phase. Under these circumstances, the Cr phase and Cu substrate had different structures and lattices, and the precipitated Cr phase had a balanced BCC structure. According to the IFFT image (Figure 2g), Cr phases were precipitated from the Cu substrate and lattice distortion was relieved significantly compared to that in the solid solution state (Figure 1c). Moreover, there were some misfit dislocations in the substrate. After ageing at 500 °C, there were undissolved phases on the grain boundaries (GBs) and some fine Cr phases precipitated in the crystals, accompanied by some activity dislocation lines near the GBs. The undissolved phases are caused by the fact that the solution degree of the alloy reaches the limit.

**Figure 3:**
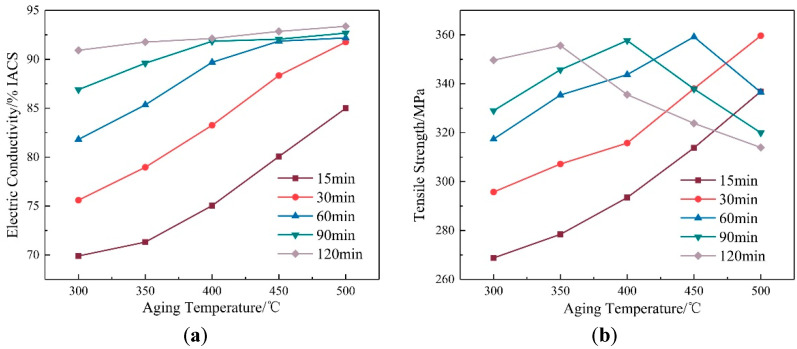
Property curves of the alloy with ageing temperature: (**a**) Electric conductivity; (**b**) tensile strength.

The variations in the tensile strength and electrical conductivity of the Cu-Cr-Zr alloy in the ageing temperature range of 300–500 °C are shown in Figure 3. Clearly, the electrical conductivity of the alloy after ageing exhibited increasing trends with increases in the ageing temperature, the tensile strength first rises and then decreases. When ageing at 300 °C for 30 min, the electrical conductivity (75.6 ± 0.8% IACS) and tensile strength (296 ± 6 MPa) of the alloy were higher than those of the alloy in the solid solution state. Moreover, the electrical conductivity increased (~119%) more significantly than the tensile strength (~15.36%). After ageing at 500 °C for 30 min, the tensile strength of the alloy was 360 ± 7 MPa and electrical conductivity was 91.8 ± 0.2% IACS, which were increased by about 40.28% and 165.97% in comparison to those of the alloy in the solid solution state.

### 3.2. Influence Laws of Holding Time on Ageing Structures

**Figure 4:**
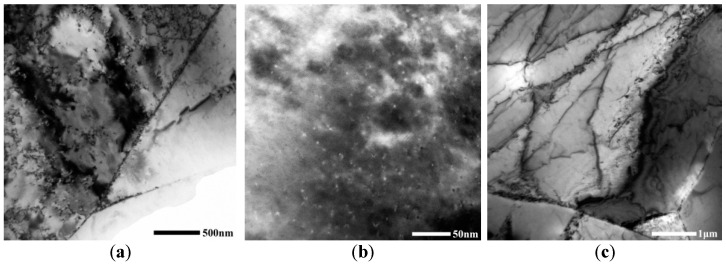

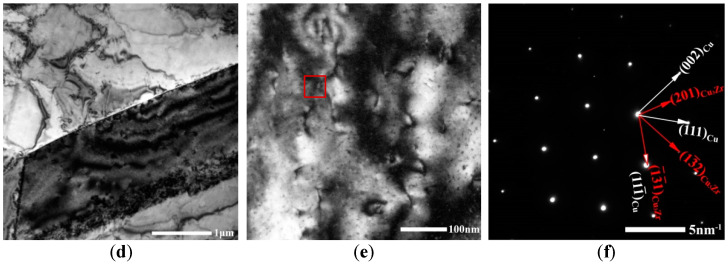
TEM images after ageing at 450 °C under different holding times: (**a**) 15 min; (**b**) 30 min; (**c**) 60 min; (**d**) 90 min; (**e**) 120 min; (**f**) SAED of the square area in (**e**).

TEM images of the solid solution state Cu-Cr-Zr alloy after ageing at 450 °C under different holding times are shown in Figure 4. With increases in the holding time, the number of Cr phases in the ageing structures increased until the completion of Cr precipitation in the supersaturated solid solution; furthermore, the size of the precipitated phases increased continuously. However, the lattice constant and dislocation density of the alloy decreased continuously (Table 2). When the holding time was 15 min, very small Cr phases were precipitated at the GBs and positions with high dislocation densities (Figure 4a). When the holding time was 60 min (Figure 4c), there were extended dislocations in the alloy structure and more phases precipitated in the substrate, which were mainly at positions with high dislocation density and on the GBs. When the holding time was 90 min, it can be seen from the TEM images (Figure 4d) that the size of the precipitated Cr phases increased. When the holding time was 120 min (Figure 4e), there were several secondary phases in the substrate, which had a dispersed distribution. According to the calibration of the SAED image (Figure 4f), there were Cu_3_Zr precipitations in the substrate, except for the secondary Cr phases.

**Figure 5:**
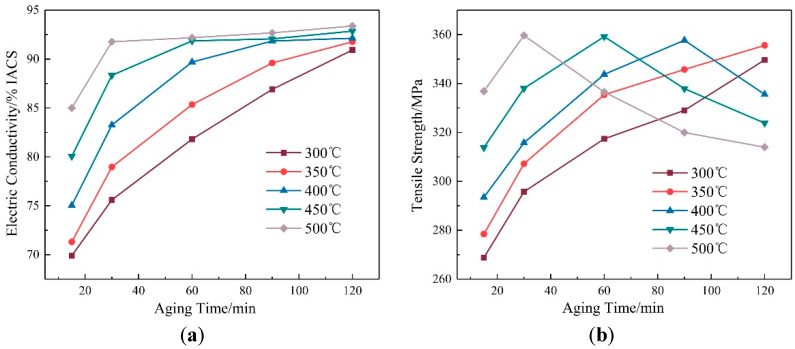
Property curves of the alloy with ageing time: (**a**) Electric conductivity; (**b**) tensile strength.

The variations in the tensile strength and electrical conductivity of the Cu-Cr-Zr alloy with holding times from 15 min to 120 min are shown in Figure 5. The tensile strength of the alloy presented an inverted V-shaped variation with the holding time. The electrical conductivity presented a rising trend and the increased amplitude in electrical conductivity decreased when the ageing temperature was fixed at 450 °C. From 15 min to 60 min, the tensile strength of the alloy increased as the amplitude decreased. From 60 min to 120 min, the tensile strength decreased with the reduction in amplitude. The electrical conductivity of the alloy continued to increase with the same trend as above. The tensile strength of the alloy reached a peak (359 ± 2 MPa) when the holding time was 60 min and the electrical conductivity was 91.9 ± 0.7% IACS.

Influenced by the free energy and diffusion energy, the secondary phases were precipitated from the oversaturated solid solution of the Cu-Cr-Zr alloy, and the nucleation grew continuously. Therefore, the ageing temperature and holding time play an important role in the precipitation of secondary phases from the alloy. According to the experimental results in this study, the precipitation, nucleation, and growth mechanisms of the secondary phases during the ageing of the alloy, as well as the influence mechanism of the structure on the properties of the alloy, were analyzed.

## 4. Discussion

### 4.1. Nucleation Mechanism of Precipitated Phase in the Cu-Cr-Zr Alloy

It can be seen from Figure 2 and Figure 3 that the FCC Cr phases were precipitated gradually during ageing from the Cu-Cr-Zr alloy after solid-solution treatment. These FCC Cr phases further evolved into BCC Cr phases. The number of precipitated Cr phases in the structures was positively related to the ageing temperature and holding time.

Cheng and Peng et al. [14,20] demonstrated that, in Cu-Cr alloys, Cr atoms exhibited an obvious segregation phenomenon (Cr-atom clustering) in the early ageing stages. Since the radius of the Cr atoms was 0.185 nm and the radius of the Cu atoms was 0.128 nm, Cr atoms may produce distortion in the Cu substrate, resulting in a sharp growth in the free energy. As ageing continued, the precipitated Cr phases nucleated and grew at the structural defects, lowering the free energies, which was attributed to the diffusion of Cr atoms under thermodynamic conditions. The structure and size changed continuously after the nucleation of the precipitated Cr phases. In other words, different sizes of precipitated Cr phases were produced on the Cu substrate under different ageing temperatures and holding times.

According to an analysis of Figure 2a, the alloy was in the early stages of ageing precipitation when the ageing time was 300 °C and the holding time was 30 min. In this stage, it was clear that no secondary phases were precipitated. However, it was found from the TEM images at the right corner that FCC Cr phases that were coherent with the Cu substrate were precipitated in the alloy. This was mainly attributed to the highly symmetrical structures of the Cu substrate and the elastic strain caused by the clustering of solute Cr atoms in the substrate [9]. In SAED, there was some secondary diffraction of BCC Cr phases and diffraction spots of the super-lattices, indicating that FCC Cr phases were transforming into BCC Cr phases in an orderly manner. This was consistent with the research conclusions of Xia and Cheng et al. [12,20]; however, in this study, no clustering of Cr atoms was observed. This was because the Zr atoms in the alloy had relatively high bonding energies and were mainly located in various structural defects like vacancies, dislocations, faults, and GBs. This promoted the segregation of Cr significantly and accelerated the precipitation of FCC Cr phases, leading to the disappearance of the GP region.

**Figure 6:**
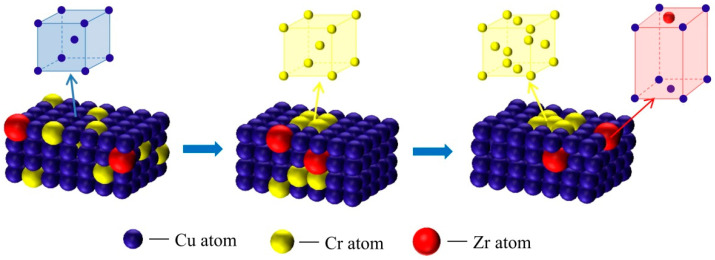
Schematic diagram of the phase precipitation.

Based on the abovementioned analysis, the precipitation order of Cr phases was as follows: Oversaturated solid solution; FCC Cr phases; BCC Cr phases, as shown in Figure 6. The orientation relationship between the precipitation of FCC Cr phases and the Cu substrate was Nishiyama–Wassermann (N–W): (110)_Cr_ // ($11\bar{1}$)_Cu_, (002)_Cr_ // ($2\bar{2}0$)_Cu_, and [$\bar{1}10$]_Cr_ // [112]_Cu_. There were several dislocations surrounding the FCC Cr phases. This is because the dislocation energies caused by lattice distortion at dislocations can facilitate the diffusion of solute atoms and concentrate at dislocations gradually, increasing the local solute concentration. As a result, the precipitated phases nucleated and grew first at positions with high dislocation densities.

The precipitation continued as the ageing temperature rose and holding time increased, which led to a growth in the percentage of the precipitated Cr phases and a continuous reduction in the nucleation quantity. This is because, as the ageing precipitation continued, Cr atoms in the alloy formed nano-secondary phase grains in oversaturated α-Cu solid solutes, the concentration of Cr solute atoms in the substrate decreases rapidly, dislocations continue to work and annihilate, the driving force for nucleation decreases, and the nucleation speed becomes slower [8]. The oversaturated solid solubility of the substrate decreased quickly and the dislocation moved and disappeared gradually. Consequently, the driving force for nucleation decreased continuously and the nucleation decelerated. Figure 2d shows that the precipitated secondary phase was the balanced BCC Cr phase when the ageing time was 450 °C and the holding time was 30 min. The orientation relationship between the BCC Cr phase and the Cu substrate was Kurdjumov–Sachs (K–S): ($\bar{1}01$)_Cr_ // ($1\bar{1}1$)_Cu_ and [010]_Cr_ // [$\bar{1}01$]_Cu_.

The results showed that Cu_3_Zr phases were precipitated in the alloy, in addition to the Cr phase (Figure 2f). This was because some Cr atoms acted on Zr atoms to form metastable CuCrZr phases during Cr precipitation in the substrate with the same orientation as the substrate, and the CuCrZr phases were then decomposed into Cu_3_Zr and Cr phases rapidly [1,6,13]. Moreover, the precipitation of abundant Cr phases from the substrate delayed the nucleation of Cu_3_Zr phases. This is different from Cu_5_Zr and Cu_4_Zr found by most researchers [24,25,26,27,28] but similar to Cu_51_Zr_14_ [33]. The different reasons may be related to the Zr content in the alloy [34]. Additionally, Cu_3_Zr maintained a K–S orientation relationship with the substrate: ($\bar{1}\bar{3}1$)_Cu3Zr_ // ($11\bar{1}$)_Cu_ and [$\bar{1}12$]_Cu3Zr_ // [$11\bar{1}$]_Cu_.

### 4.2. Growth Mechanism of Precipitated Phases in the Cu-Cr-Zr Alloy

The nucleation and growth of secondary phases in the Cu-Cr-Zr alloy occurred simultaneously. With increases in the ageing temperature and holding time, the precipitated phases began to grow continuously after nucleation and the energies for the growth of phases increased when the nucleation decreased during ageing precipitation. The growth of precipitated Cr phases and the migration of the Cr phase-Cu substrate interface both required the diffusion of atoms. Hence, the growth of the precipitated phases in the alloy can be used to interpret the diffusion theory.

In the early ageing stages, the FCC Cr phases maintained a coherent relationship with the Cu substrate. Although the interface between the FCC Cr phases and the Cu substrate exhibited very small energy differences, the alloy had considerably high interface energies due to the nucleation of many FCC Cr phases, which increased the instability of the structure. To decrease the interface energies, the FCC Cr phases clustered and grew in the manner of the growth of big grains and the dissolution of small grains. FCC Cr phases tend to grow spherically due to isotropic distortion and they continue to become increasingly spherical [1,24,35]. In the growth process of the precipitated Cr phases, the Cr atom concentration in the adjacent Cu substrate varies due to the different sizes of the Cr phases. Hence, there were concentration gradients among different sizes of Cr phases in the Cu substrate. Cr atoms diffused from the areas surrounding small-sized Cr phases to areas surrounding large-sized phases [36]. This facilitated the growth of large-sized Cr phases and the shrinking and dissolution of small-sized ones. The degree of growth in the Cr phases was determined by the diffusion rate of the Cr atoms. The Cr atoms diffused more quickly at a higher temperature and the degree of growth in the Cr phases increased. When the temperature was fixed, the diffusion rate of Cr atoms was constant. As the holding time increased, the degree of growth in the Cr phases became constant. Therefore, the Cr phases in the alloy grew gradually with increases in the temperature and holding time, thus increasing the lattice distortion energies. When the Cr phases and lattice distortion energies increased to a certain extent, the Cr phase-Cu substrate interface developed coherent buckling. The interface relationship changed to semi-coherent and finally changed to non-coherent.

### 4.3. Influence Laws of Cu-Cr-Zr Alloy Structure on Its Properties

According to an analysis of Table 1 and Table 2, nano Cr phases were precipitated from the oversaturated solid solution when the ageing temperature and holding time increased. In addition, the solute atom content in the substrate decreased. Furthermore, the dislocations moved and disappeared gradually. The precipitation and growth of the secondary phases could hinder the movement of dislocations, thus decreasing the dislocation density and lattice distortion in the alloy.

In the early ageing stages, the dislocation density and lattice constant of the alloy were negatively related to the ageing temperature and holding time; hence the electrical conductivity of the alloy was positively related to the ageing temperature and holding time, while the tensile strength was negatively related to them. The number of precipitated fine secondary phases that were coherent with the Cu substrate increased continuously and the degree of supersaturation of the solid solution decreased sharply. As a result, the electrical conductivity and tensile strength of the alloy improved accordingly. The dislocations interacted with the precipitated phase, which increased the diffusion rate of the solute atoms in zones with high dislocation densities and facilitated the precipitation of Cr phases. The Cu substrate-Cr phase interface might hinder the dislocation movement and pin dislocations [37]. The precipitated phase influenced the tensile strength significantly more than the dislocations [10,11,35]. After the ageing treatment at 300 °C for 30 min, the dislocation density and lattice constant in alloy structures were 2.439 × 10^15^ m^−2^ and 0.42338 nm, respectively, which were 3.5% and 6.8% lower than those in the solid solution state, respectively. Moreover, the tensile strength (296 ± 6 MPa) and electrical conductivity (75.6 ± 0.8% IACS) were 15.36% and 119% higher than those in the solid solution state, respectively. Since the precipitate phases had very small content, the tensile strength was improved to a very small extent. Similarly, the dislocation density and lattice constant in the alloy structures were 2.376 × 10^15^ m^−2^ and 0.38925 nm after the ageing treatment at 450 °C for 15 min, respectively, which were 6% and 14.35% lower than those at the solid solution state, respectively. Moreover, the tensile strength (314 ± 3 MPa) and electrical conductivity (80.1 ± 0.5% IACS) were increased by about 22.40% and 132.06% compared to those before the ageing treatment, respectively.

The ageing precipitation continued with the continuous increase in the ageing temperature and holding time. Therefore, the dislocations in the alloy structure migrated and disappeared continuously, and the secondary phases were further precipitated. The lattice constant and dislocation density of the substrate continued to decrease. The coherent relationship between the secondary phase and Cu substrate in the alloy changed to a semi-coherent relationship gradually. Electron scattering was relieved by decreasing the solid solution in the substrate [12,13,14], thus increasing the electrical conductivity and tensile strength of the alloy gradually. Hence, the lattice constant and dislocation density of the alloy decreased to 0.38714 nm and 2.051 × 10^15^ m^−2^ after an ageing treatment at 450 °C for 30 min, respectively, while the tensile strength and electrical conductivity increased to 338 ± 4 MPa and 88.3 ± 0.9% IACS, respectively. The lattice constant and dislocation density of the alloy decreased to 0.37517 nm and 1.639 × 10^15^ m^−2^ after an ageing treatment at 500 °C for 30 min. Under these conditions, the lattice constants of the alloy and the pure Cu were very similar [1,9,36]. The tensile strength and electrical conductivity increased to 360 ± 7 MPa and 91.8 ± 0.2% IACS, respectively.

**Figure 7:**
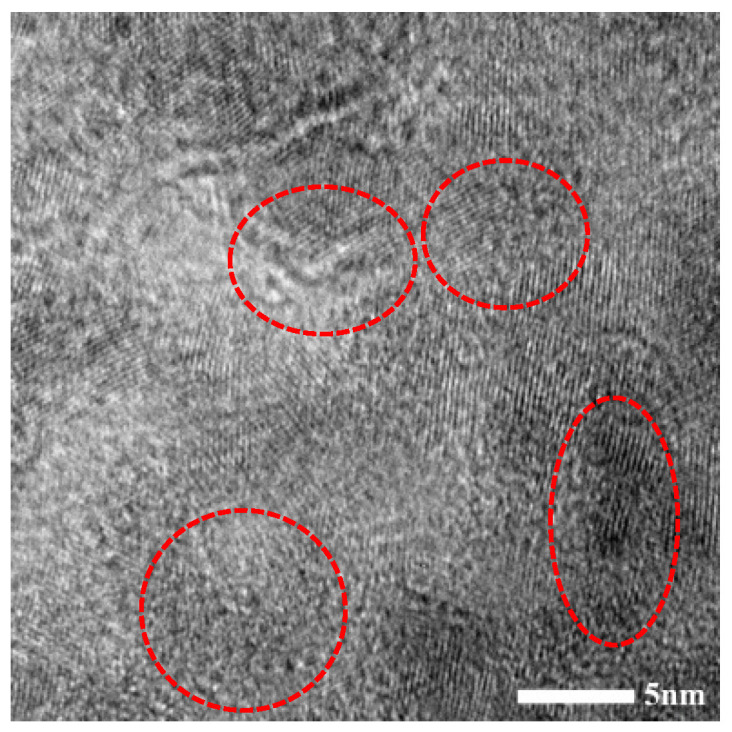
HRTEM image of the alloy after ageing treatment at 450 °C for 60 min.

Solute atoms almost completely precipitated at the ageing peak. The electron scattering was very small and the tensile strength was improved to the highest extent by the precipitated phase. According to Figure 4 and Figure 7, there are many elliptical precipitates in the alloy and they remain semi-coherent with the substrate after the ageing treatment at 450 °C for 60 min. Influenced by Zr, more Cr phases were precipitated at positions with high dislocation densities and GBs. Since the stacking fault energy [38,39] of the alloy was relatively low, it is difficult to incur helical dislocation slippage surrounding the precipitated Cr phases. Perfect dislocations were decomposed into two or several partial dislocations; therefore, there are extended dislocations in the structures. Due to structural changes, the tensile strength and electrical conductivity of the alloy were 359 ± 2 MPa and 91.9 ± 0.7% IACS, respectively.

As the ageing treatment continued, the size of the precipitated Cr phases in the alloy increased gradually, whereas the lattice constant and dislocation density decreased. Both led to reductions in the tensile strength and growth in the electrical conductivity of the alloy. Since the lattice constant and dislocation density had very small influences on the electrical conductivity, it increased slightly. After the ageing treatment at 450 °C for 90 min, the lattice constant and dislocation density of the alloy decreased to 0.37191 nm and 2.376 × 10^15^ m^−2^, respectively. Under these circumstances, the tensile strength of the alloy decreased to 338 ± 9 MPa, while the electrical conductivity continued to increase to 92.1 ± 0.1% IACS. After the ageing treatment at 450 °C for 120 min, the tensile strength continued to decrease to 324 ± 4 MPa, while the electrical conductivity increased to 92.9 ± 0.6% IACS.

## 5. Conclusions

In this study, the precipitation behavior and property changes of the Cu-Cr-Zr alloy under different ageing conditions were discussed. Some major conclusions can be drawn, as follows:

1. With the increase in ageing temperature and holding time, the percentage of Cr precipitated phase in the microstructure increases, and the dislocation content decreases continuously. The tensile strength increases first and then decreases in an inverted V shape. The electrical conductivity shows a rising trend with then a decrease of the increase;
2. The tensile strength of the Cu-Cr-Zr alloy reached a peak (359 ± 2 MPa) and the electrical conductivity was 91.9 ± 0.7% IACS after the ageing treatment at 450 °C for 60 min;
3. The precipitated Cr phase has two structures, FCC and BCC. The FCC Cr phases are transformed into BCC Cr phases. The FCC Cr precipitated phases nucleate first in regions with high dislocation densities, basically maintaining an N–W orientation relationship with the substrate. The BCC Cr phases basically maintain a K–S orientation relationship with the substrate. The precipitated Zr phase is the Cu_3_Zr phase, which basically maintains a K–S orientation relationship with the substrate.

## Figures and Tables

**Table 1:** Lattice distortion and dislocation density after 30 min ageing at different temperatures.

**Ageing Temperature/** **°C**	300	350	400	450	500
**Lattice Constant/nm**	0.42338	0.41083	0.39517	0.38714	0.37517
**Dislocation Density/** **(** **10^15^·m^−2^** **)**	2.439	2.385	2.185	2.051	1.639

**Table 2:** Lattice distortion and dislocation density after ageing at 450 °C under different holding times.

**Ageing Time/min**	15	30	60	90	120
**Lattice Constant/nm**	0.38925	0.38714	0.37604	0.37191	0.36204
**Dislocation Density/** **(** **10^15^·m^−2^** **)**	2.376	2.051	1.910	1.376	1.132

## Data Availability

The data used to support the findings of this study are available from the corresponding author upon request.
